# Prediction of Human Performance Using Electroencephalography under Different Indoor Room Temperatures

**DOI:** 10.3390/brainsci8040074

**Published:** 2018-04-23

**Authors:** Tapsya Nayak, Tinghe Zhang, Zijing Mao, Xiaojing Xu, Lin Zhang, Daniel J. Pack, Bing Dong, Yufei Huang

**Affiliations:** 1 Department of Electrical and Computer Engineering, University of Texas at San Antonio, San Antonio, TX 78249, USA; ani254@my.utsa.edu (T.N.); prh169@my.utsa.edu (T.Z.); mzj168@hotmail.com (Z.M.); 2 NSF-DOE CURRENT Center, University of Tennessee, Knoxville, TN 37996, USA; xiaojing.hsu@gmail.com; 3 SIEE, China University of Mining and Technology, Xuzhou 221116, China; cnnangua@hotmail.com; 4 College of Engineering & Computer Science, University of Tennessee, Chattanooga, TN 37403, USA; daniel-pack@utc.edu; 5 Department of Mechanical Engineering, University of Texas at San Antonio, San Antonio, TX 78249, USA; bing.dong@utsa.edu

**Keywords:** human performance, performance prediction, indoor room temperature, office-work tasks, electroencephalography (EEG)

## Abstract

Varying indoor environmental conditions is known to affect office worker’s performance; wherein past research studies have reported the effects of unfavorable indoor temperature and air quality causing sick building syndrome (SBS) among office workers. Thus, investigating factors that can predict performance in changing indoor environments have become a highly important research topic bearing significant impact in our society. While past research studies have attempted to determine predictors for performance, they do not provide satisfactory prediction ability. Therefore, in this preliminary study, we attempt to predict performance during office-work tasks triggered by different indoor room temperatures (22.2 °C and 30 °C) from human brain signals recorded using electroencephalography (EEG). Seven participants were recruited, from whom EEG, skin temperature, heart rate and thermal survey questionnaires were collected. Regression analyses were carried out to investigate the effectiveness of using EEG power spectral densities (PSD) as predictors of performance. Our results indicate EEG PSDs as predictors provide the highest *R*^2^ (> 0.70), that is 17 times higher than using other physiological signals as predictors and is more robust. Finally, the paper provides insight on the selected predictors based on brain activity patterns for low- and high-performance levels under different indoor-temperatures.

## 1. Introduction

As U.S. citizens spend more than 90% of their time indoors, indoor thermal condition is a key factor that impacts human productivity in the office [1,2,3,4,5]. Indoor environments and building characteristics have been reported to impact occurrences of respiratory diseases, allergy and asthma symptoms, sick building symptoms and office-work performance. It is estimated that improving the indoor environment in U.S. office buildings would result in a 0.5 to 5% increase in productivity, worth $12–$125 billion annually [6]. Thus, understanding how indoor environments affect human performance, health and emotion and developing methods to predict human performance/health in changing indoor environments have become highly important research topics that bear significant economic and sociological impact. 

As our indoor daily work becomes increasingly mentally challenging, a significant aspect of the thermal-driven performance is an individual’s cognitive performance, that is, the ability of an individual to effectively comprehend and perform independent decisions during complex tasks and events. Various field and laboratory studies have been conducted to investigate performance levels and changes under different thermal conditions. A study investigated in Reference [7] showed an 8% fall in sewing work productivity as indoor temperature was increased from 23.9 °C to 32.2 °C. A similar trend was observed in a case study by References [8,9] investigating the performance of employees in telecommunication offices (call center) and a reported decline in work performance by 5–7% at higher indoor temperatures; work performance was evaluated by assessing average time per call or average handling time. Similar studies were conducted to evaluate the performance of school children in References [10,11]. In the former research study, students who reported changes in thermal sensation scores from warm to neutral, performance of numerical and language task improved significantly, while the latter concluded thermal stress produces mental arousal effects thereby improving performance. In addition to these papers that studied the influence of indoor environment on office work performance, researchers have investigated physiological mechanisms and whether these mechanisms have consequences for human performance. At high temperatures, authors in Reference [12] reported that the concentration of carbon-dioxide (CO_2_), by measuring end-tidal partial CO_2_, is directly proportional to the increase in room temperature, which they hypothesize is the result of increased metabolism by humans in turn leading to decreased air quality. Furthermore, they observed a reduction in arterial blood oxygen saturation (SPO_2_), increasing sick building syndrome (SBS) symptoms thereby elevating fatigue levels in participants. A brain imaging near-infrared spectroscopy (NIRS) study by the authors in Reference [13] observed a reduction in task performance as blood oxygen saturation levels decrease. Interestingly, while Reference [14] found decreased concentrations of salivary alpha-amylase and cortisol with increased thermal discomfort—implying an impact on performance—but performance did not change. On the other hand, they found carbon dioxide concentrations to be similar at different indoor temperatures thereby suggesting no change in metabolic rate, however subjects reported significant increase in workload and effort with increased thermal discomfort. Other detailed research in Reference [15] studied the effects of cold temperature on cognitive performance, wherein they observed three distinct performance patterns—negative, positive and mixed, which were determined based on accuracy, response time and efficiency based on a cognitive test battery. They concluded that skin temperature, thermal sensation, diastolic blood pressure and heart rate were independent predictors of decreased accuracy and response time and concluded that cold temperatures impact performance negatively due to mechanisms of distraction and arousal. These past studies indicate performance trends change depending on the task and environmental conditions, which is not always straightforward. More research evidence suggests that human performance is a byproduct of psychological and physiological factors collectively, which we theorize may be better explained by neurophysiological signals.

Taking into account the relationship between human performance and indoor thermal conditions and the advantages of predicting performance by potential improvements on office-workers’ health and productivity, we propose to use neurophysiological signals from electroencephalography (EEG) as predictors of performance. Over time, EEG research has been extensively used and shown to be effective in the detection and interpretation of brain mental states during the execution of cognitive and physical tasks. Specifically, the association of cognitive functions with specific brain regions and their temporal characteristics have been determined from imaging studies such as functional magnetic resonance imaging (fMRI), evoked response potential (ERP) and time-frequency analyses of EEG or EEG-MEG (magnetoencephalography) studies. Working memory studies have shown theta band (4–8 Hz) power is correlated with cognitive performance; high-performing individuals or individuals with working memory training exhibit increased theta power in the frontal-parietal brain network [16]. Studies in References [17,18,19,20,21] have also reported the functional involvement of the frontal-parietal network associated with working memory and executive functions and References [22,23] have reported the involvement of the frontal-temporal declarative and semantic memory network associated with controlled retrieval of task-relevant facts or rules. Researchers have also analyzed the temporal dynamics of these networks; time-frequency analysis in Reference [17] during arithmetic problem-solving tasks shows the engagement of the frontal cortex at around 300 ms from stimulus presentation for memory retrieval strategies reflected as enhanced theta power within the frontal-temporal network. On the other hand, procedural strategies have higher execution demands at later time points, reflected as alpha power event-related desynchronization (ERD) in the frontal-parietal networks. 

Analogous to arithmetic problem-solving tasks, brain dynamics have also been reported in tasks involving motor movements where focused attention and somatosensory information processing play a crucial role [24,25,26]. Tasks that involve motor movements are associated with the activation of contralateral sensorimotor cortex, where findings by References [27,28,29] report an increase in theta power localized at the fronto-midline during the onset or preset of a motor movement particularly during high performance or by expert performers and increased theta power was additionally observed during higher workloads. Beta (14–30 Hz) oscillations have been known to be associated with voluntary movements, particularly, beta modulations post-movement synchronization over the sensorimotor cortex has been linked to greater confidence in the execution of motor tasks suggesting reinforcement of the current motor state and generation of the steady motor output [30,31,32,33]. Beta modulation has additionally been linked to reaction time where a decrease in beta was observed upon committing an error resulting in longer reaction times for upcoming trials due to increased cognitive load [34].

Based on the evidence stated above, establishing performance changes under varying environmental conditions and linkage between behavioral changes/performance with underlying brain activities, we propose to use EEG brain signals to predict performance. With this goal, we present an experimental design wherein subjects perform mental tasks under varying thermal conditions and develop linear regression models to predict performance using EEG power spectral densities (PSD) as features/regressors. Specifically, we theorize the involvement of theta power from the frontal-temporal or frontal-parietal network in arithmetic problem-solving and the involvement of theta and beta/alpha power band from the fronto-midline and motor-cortex for typing tasks to vary at different performance levels. Both office-work tasks in this study require crucial physiological factors such as sustained attention, working memory, self-motivation and motor control specific to typing tasks. To achieve our goal, we first compute the prediction strength of features such as thermal survey scores, heart rate and skin temperature and then compare them to prediction accuracies using EEG power spectral densities from linear regression models. Given the spatial-temporal brain dynamics to complete the task, we implement least absolute shrinkage and selection operator (LASSO) as a feature selection technique to select relevant power densities from brain regions contributing towards explaining performance. Lastly, the robustness of these regressors is compared with other non-neurophysiological signals by reporting least mean square errors (MSE).

## 2. Materials and Methods

### 2.1. Office-Work Task Simulation

All participants were required to complete two types of office work task—addition and typing—in two different indoor room temperatures, 22.2 °C (72 F) or 30 °C (86 F). Each task lasted for 15 min (30 min in total). The difficulty level of each task ranged from easy to average, designed with the intention of simulating daily office responsibilities. All participants were provided with a training session to familiarize themselves with the experiment setup, task instructions and software interface. 

All participants attempted the addition task first, involving the addition of two three-digit numbers, which were generated randomly online in MATLAB [35]. The task was designed to be self-paced and participants were instructed to avoid errors while attempting as many questions as possible in 15 min; thus, the total number of questions answered by each participant depends on their response time for each question. This was followed by 15 min of a typing task, in which all participants were instructed to type the paragraph (4 sentences long) exactly as presented on the display monitor and was self-paced. The writing paragraph for this task was selected from a journal and no limit was posed on the number of paragraphs typed—that is, every time the participant finished typing the current paragraph, a new paragraph was presented. Similar instructions were provided, that is, to avoid any typing errors and to attempt typing as many paragraphs as possible. The typing software continuously monitored the typed words for errors, in which case the participant had to correct them before proceeding to the next word. Contrary to the typing task, wherein the participant is aware of typing errors and must correct them, in the addition task the participant is unaware of their response accuracy, that is, no feedback was provided. 

MATLAB [35] was used to design and program the addition task presentation and the typing task software was developed by the National Research Council of Canada [36].

### 2.2. Participants

Seven healthy male adult participants, all university students, were recruited for this study whose age ranged between 18 and 25 years (mean age = 23.5 ± 0.8 years). All provided written consent to participate in the study, which was approved by the Institution Review Board at the University of Texas, San Antonio and stated that they were healthy, without any neurological issues and were not under the influence of any drugs at the time of the experiment. All participants reported to have at least five hours of sleep the night before the experiment and dressed in formal casuals (jeans with long sleeve shirts) for the experiment. This clothing level was selected to keep the participants thermally neutral at room temperature 22.2 °C (72 F), which is reported as a neutral temperature to achieve optimal performance. The study was conducted in an experiment room simulating an office environment with comfortable lighting. Each participant was exposed to two thermal conditions—22.2 °C (72 F) and 30 °C (86 F). A ventilation rate of 6 L/s per person was kept constant at both room temperatures and the relative humidity in room was maintained within normal recommended limits. Lastly, before beginning the experiment all participants were instructed to focus and not to move their head or talk during the task.

### 2.3. Experimental Procedure

**Figure 1:**
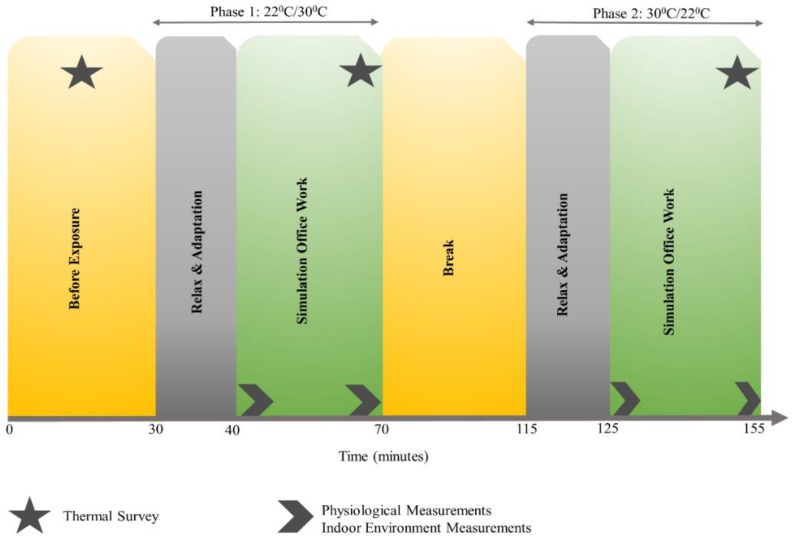
Illustration of experiment timeline.

First, all participants were guided to a preparation room where a neutral temperature of 22.2 °C was maintained. Here, participants were prepped for the experiment, that is, sensors for measuring skin temperature, heart rate and the EEG cap were attached. After which, they were guided to the experiment-room, the room temperature was randomly maintained at either 22.2 °C or 30 °C, see Figure 1. All participants were seated on a comfortable chair 50 cm away, from the center of the monitor to the participant’s eye. Before the start of the first office-work task under each exposure (or session), 10 min of rest time was provided to adapt to the thermal settings and all participants were alone in the experiment-room. Prior to the second exposure, a 45-min break was given to relax, drink water, walk around and use the restroom. In the meantime, the temperature of the experiment-room was increased or decreased depending on the temperature setting used in the first exposure. The order of the indoor room temperature was randomized for each participant, wherein 4 participants were first exposed to 22.2 °C and the remaining three participants to 30 °C. In the second session, all participants repeated the office-work task for the next 30 min. Additionally, before and after each session and each task, participants answered a short thermal survey. The entire experiment lasted for 155 min.

### 2.4. Measurements

#### 2.4.1. Performance Metrics

All participants performed the addition and typing task for 15 min under each exposure. Response time and accuracy are the performance metrics commonly used for the addition task. To assess the overall performance in this task, the two metrics were integrated, that is, the time taken to complete 20 questions correctly, that is Equation (1):

(1) $$\begin{array}{c} Addition\;Performance\;Index\;\left(API\right)\\ \;=Time\;taken\;\left(seconds\right)\;to\;answer\;20\;questions\;correctly \end{array}$$

To increase the number of samples, a sliding window of 20 correct questions with a shift of one question is applied, moving along the dimension of number of questions answered. For instance, if the first 20 questions are all answered correctly, then the API for the first sample is calculated as the sum of the response times for answering the first 20 questions. Now, if question number 21 is incorrect but number 22 is correct, then the API of the second sample is calculated as the sum of the response times for answering questions 2 to 22 including exactly 20 correctly answered questions. Thus, for committing an error, a penalty in time is issued in the metric API. We chose 20 correct questions in the metric because most participants take approximately one minute to answer 20 questions, thus making API a stable metric to assess addition performance.

The metric used to evaluate the typing task performance is net characters per minute (CPM) [20], which is calculated as Equation (2):

(2) $$\begin{array}{c} \mathrm{Net}\;\mathrm{characters}\;\mathrm{per}\;\mathrm{minute}\;\left(\mathrm{CPM}\right)\\ \;=\mathrm{Total}\;\mathrm{number}\;\mathrm{of}\;\mathrm{key}\;\\ \;-\left(\mathrm{Total}\;\mathrm{cursor}\;\mathrm{keys}\;\mathrm{pressed}+2\times \mathrm{Number}\;\mathrm{of}\;\mathrm{backspace}\;\mathrm{keys}\;\mathrm{pressed}\right) \end{array}$$

During the task, the user types the paragraph displayed on the screen. The text is confirmed after each word and in the case of errors a strikethrough is notified on the screen from the point of error occurrence. The user is unable to continue typing until the error has been rectified. The user is unable to use the mouse, however can move around the screen using cursor keys and can delete using BACKSPACE or DELETE keys. The typing performance metric is calculated as the net number of characters typed per minute as shown above. The backspace key is doubled as characters typed are deleted and then retyped. Thus, as the typing errors increase, the number of characters typed per minute (or typing performance) decreases. To calculate CPM samples, a sliding window of one minute was applied with a shift of 30 s.

#### 2.4.2. Physical Measurements

The temperature and relative humidity of the experiment-room were continuously maintained and recorded with data loggers—temperature (range: 20 °C to 70 °C, accuracy: ±0.7 °C), humidity (range: 0–95%, accuracy: ±5%) and CO_2_ (range: 0–2000 ppm, accuracy: ±50 ppm) sensors. All sensors were calibrated before use.

Subjective measurements: A survey/questionnaire was provided to all participants before each task to assess the room thermal conditions (comfort and sensation) and air-quality. The perceived thermal comfort and sensation conditions were assessed using continuous scales describing participants’ satisfaction in the thermal environment. In case of thermal comfort, participants reported their comfort level in the room temperature under an exposure. A score of one-point indicates very uncomfortable, 4-point indicates just right and seven-point indicates very comfortable. Likewise, for thermal sensation, one-point indicates cold, 4-point indicates neutral and seven-point indicates hot body sensation. In addition to these questions, participants also answered questions indicating their general indoor thermal preference and if they preferred the current room temperature to be changed.

#### 2.4.3. Physiological Measurements

The physiological measurements included: (1) skin temperature measured from eight sensors located at forehead, right scapula, left upper chest, wrist, both upper arms, left hand, left-calf and right anterior thigh according to ISO 9886 standards. Samples were recorded every second and for analysis purposes a weighted average skin temperature was computed, recommended by ISO 9886 standards [37]; (2) Heart rate was measured by using Polar H7 Smart Chest Transmitter (Polar Electro Oy, Kempele, Finland) and recorded on an iPad via Bluetooth every second.

#### 2.4.4. EEG Measurement and Preprocessing

Brain activities were continuously recorded at a sampling rate of 512 Hz using 64-channel EEG system (Biosemi, Inc. [38]) referenced to the right and left ear mastoids based on a modified international 10–20 system. Before data acquisition, care was taken to ensure that the impedance between EEG electrodes and cortex was less than 5 kΩ. From each participant, 30-min EEG signals during each exposure were recorded and preprocessed prior to obtaining power spectral density (PSD) values for further analysis. EEG preprocessing involved down-sampling the data to 128 Hz, bad channel removal and interpolation using the software EEGLab [39], referencing each EEG electrode using the average signal from left and right ear mastoid connections, bandpass filtered between 1 and 50 Hz to remove electrical noise, DC shift and artefact removal introduced by eye blinks and muscle movements. EEG data from each participant from both exposures were normalized using *z*-scores. Preprocessing was followed by average PSD value computation for each EEG electrode data epoch. Length of the epochs depended on the type of office work task metric, for the addition task, the length of epochs was based on the time taken to answer 20 questions correctly from its metric API and for the typing task, an epoch length of one minute was extracted based on its metric net CPM. To increase the sample size, a sliding window was applied, wherein for the addition task, a sliding window of 20 questions with one question shift was applied and for the typing task, a sliding window of one minute with a 30-min shift was applied.

## 3. Results and Discussion

The goal of this paper is to assess the efficiency of using EEG signals in performance prediction induced by varying indoor room temperatures. To do so, this paper is organized into three parts: first, we present statistical results to validate performance is effected by indoor temperatures; second, we show the prediction results of office-work performance using features reported by past research—thermal sensation, thermal comfort, skin temperature and heart rate in a linear regression model; and third, we present the prediction ability and robustness of using EEG PSDs as predictors in a linear regression model enhanced with LASSO. 

### 3.1. Performance versus Room Temperatures

Table 1 and Table 2 summarize the statistical test results of all seven subjects during each office-work task to determine change in performance under different indoor temperatures. For each task, the average performance of the corresponding task is reported under each temperature exposure along with standard deviation in parentheses and respective *p*-values. Additionally, prior to and after the experiment, all participants answered a thermal survey reporting their comfort levels at 22.2 °C and 30 °C and most felt comfortable at 22.2 °C, which is considered the control exposure in our study design.

The Kolmogorov-Smirnov (KS) statistical test was used because performance values for both office-work tasks did not follow a normal distribution. In the addition task, the samples used for the KS-test were the time taken to answer 20 questions correctly with a sliding window with an overlap of 19 questions, thus low performance corresponds to a longer time taken to answer 20 questions. The test revealed that all seven participants showed significant differences in performance between the two exposures (*p*-value < 0.1). As expected, we observed that four out of seven participantsshowed low performance at elevated temperature of 30 °C. In theseparticipants, an increase in response time to answer the arithmetic problems could be attributed to fatigue thereby requiring higher cognitive demand. In the typing task, KS-test samples used were the net characters typed per minute with a sliding window of one minute with an overlap of 30 s, thus fewer characters per minute reflects low performance. Five participantsout of seven showed significant differences in performance between the two exposures, among whom, interestingly, four subjects performed higher in the elevated room temperature of 30 °C. Based on participantfeedback, this is attributed to discomfort at the elevated temperature and thus wanting to finish the task quickly. Although we would expect to observe an increase in typing errors with increased typing speed, this was perhaps not the case because the task was self-paced. Based on these statistical results, we can conclude that indoor room temperature affects office-work performance and increaseor decrease of performance under different room temperatures is task dependent. 

### 3.2. Performance versus Physiological Signals

Table 3 shows the correlations between office-work performance using features reported by past research groups, that is, from thermal survey votes (thermal sensation, thermal comfort) and physiological recordings (skin temperature and heart rate). Data samples used to compute correlations (*R*^2^) between performance and physiological recordings during both tasks are as described in Section 2.4, with the implementation of sliding window for all participants. To compute correlations with thermal sensation and comfort, survey scores were collected at the end of each office-work task (see Figure 1) and corresponding average task performance from all participants were used, without sliding window. Empirical *R*^2^ results show that all predictors exhibit a correlation less than 0.5 ranging between 0.003 and 0.1, indicating that each individual regressor is unable to explain variance in office-work performance and do not exhibit a linear trend. Past research studies have reported correlations of heart-rate variability with mental effort due to its association with blood pressure regulation [40,41]; however, linear correlation analysis in this case did not show significant *R*^2^ correlations. Due to the small and elusive nature of *R*^2^ values, we proceed to investigate the correlation of office-work performance using brain signals obtained from EEG. 

### 3.3. Performance versus EEG 

To investigate the efficiency of predicting performance using EEG power spectral densities in linear regression analysis, we first present results using brain spectral densities as features (i.e., theta, alpha, beta and combined brain bands) from each EEG electrode location separately and then present prediction ability by using a variable selection technique—LASSO—that determines the most relevant features across brain regions.

#### 3.3.1. Band Powers of Raw EEG Data as Regressors

Based on the motivations mentioned above to use EEG brain PSDs as features, we investigate the average spectral powers corresponding to these well-studied oscillations in theta band (4–8 Hz), alpha band (8–14 Hz) and beta band (14–30 Hz). 

**Figure 2:**
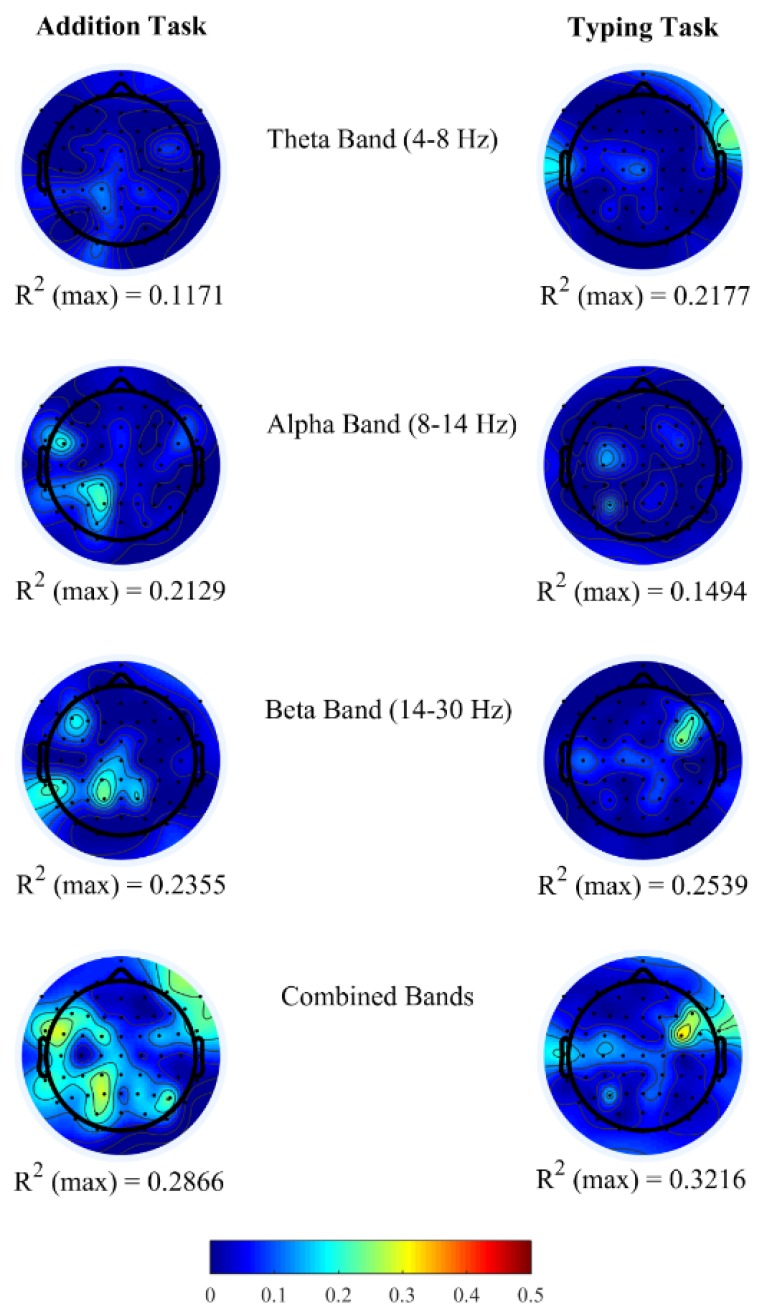
The topoplots represent the correlation *R*^2^ maps between the brain power spectral densities and office task performance.

To study the correlations between change in office-work task performance and EEG spectral bands, a linear regression *R*^2^ analysis was used to determine the relationship between performance and each EEG PSDs from each of the 64 EEG electrode locations from all participants. Figure 2 shows the topoplots of *R*^2^ values obtained for each channel from the above-mentioned three frequency bands. In both office-work tasks, we observed insufficient *R*^2^ ranging approximately between 0.10 and 0.25 when brain power bands are used as regressors individually. Furthermore, we investigated the correlation coefficients (*ρ*) between pairs of brain power bands corresponding to channels with maximum *R*^2^ in the single regressor linear model. From Table 4, we observe that the correlation coefficients between two regressors is insufficient (<0.9) demonstrating that they do not have a strong correlation, therefore denoting that each PSD contributes independently towards performance prediction. Based on this finding, it is possible to achieve higher correlations *R*^2^ by combining all three brain power bands within each EEG channel to generate a new multiple regressor linear model. In doing so, we observe a maximum correlation of *R*^2^ = 0.2866 and *R*^2^ = 0.3216 in the addition and typing tasks, each of which is an increase of 21.70% and 26.66% compared to the highest *R*^2^ using a single regressor linear model from each EEG channel. The *R*^2^ topoplots show maximum correlation in the left parietal and occipital brain regions in the addition task and in the right fronto-temporal brain regions in the typing task. 

To ensure the maximum correlation observed is not due to chance/noise permutation, the test was performed by randomizing epochs across EEG channels. *p*-value = 0 (<0.05) was obtained for all band power regressors, individually and combined, from EEG channel locations corresponding to its maximum *R*^2^ from the linear regression models. Analysis thus far supports the notion that there are multiple brain regions contributing towards an explanation of performance and helping to achieve higher prediction. With this motivation to achieve higher prediction power, we proceed to using LASSO (least absolute shrinkage and selection operator) to select relevant brain bands from specific EEG channels collectively in the next section. 

#### 3.3.2. LASSO with Brain Band Power in All Brain Regions as Regressors

To determine the subset of EEG spatial locations that collectively contribute to predicting performance, the LASSO regression analysis method was implemented. This method fits a sparse linear regression model that performs both feature selection to avoid multicollinearities and overfitting regularization to improve prediction accuracies and interpretability of statistical models. Specifically, let ***y*** ϵ ℜ^N×1^ represent a vector of performance values from *N* epochs (*N* = 2546 for addition task and 330 for typing task) and ***X*** ϵ ℜ^N×M^ be a matrix of power values of a frequency band, whose nm^th^ element denotes the power of epoch *n* at channel *m* (note when all brain bands are combined *M* = 64 × 3 = 192). LASSO fits a linear model between ***y*** and **X** given by Equations (3) and (4):

(3) $$y=X\beta +\beta_{o}+\varepsilon$$

where ***β*** ϵ ℜ^M×1^ and *β_o_* are model coefficients and ε is the *N* × 1 noise vector with zero mean and constant variance. LASSO aims to find estimates of the coefficients $\hat{\beta }$ by optimizing

(4) $$\underset{\beta_{o},\;\beta }{\min}\frac{1}{N}||y-X\beta -\beta_{o}||\;+\;\lambda \left|\beta \right|$$

where |.| and ∥.∥ denote the l_1_-norm and l_2_-norm respectively and λ is the regularization parameter [42]. The l_1_-norm constraint (2) forces the coefficients to be sparse, that is, only small subsets of coefficients are nonzero. There are two advantages of using LASSO that generate sparse constraints. First, as the dimension of the matrix ***X*** in (1) is *M* = 64 or 192, representing the number of EEG channels when PSDs are used individually and combined and *N* = 2546 and 330 performance samples from the addition and typing tasks, LASSO avoids overfitting. Secondly, the sparse coefficients make the model more interpretable, as the model focuses only on the powers from channels with nonzero coefficients. To further reduce the overfitting during model fitting, 20% of data samples were set aside for testing—called holdout data—and the remaining was used for model training. LASSO iteratively generates models with different regularization parameters on the training data, after which holdout data is used to determine a model that gives the lowest mean square error between the observed and predicted performance. To estimate the prediction ability of chosen model, *R*^2^ is computed between the observed and estimated office-work performance.

Table 5 summarizes the correlation results between observed and estimated office-work performance from the features selected by LASSO regression model for each office-work task, including the number of EEG electrodes selected by LASSO and *p*-value corresponding to the statistical significance of the model chosen. Overall, the resulting LASSO models are relatively sparse, exhibiting *R*^2^ in the range of 0.64–0.89 and, as expected, is two times greater than the maximum *R*^2^ obtained from the linear regressor model, that is, without combining spatial information, from both office-work tasks. In the addition task, we observe that all PSD regressors, individual and combined, provide correlations >0.5, with the highest *R*^2^ observed using alpha power as the regressor individually and combined with other power bands, using LASSO, at 88.6% from 51 electrode locations and 83.4% from 174 electrode locations. On comparing both *R*^2^ values, using alpha power alone, the LASSO model outperforms the latter by ~5% with contributions from fewer EEG channels (51 EEG channels), maybe by removing channels involved in multicollinearities. In the typing task, *R*^2^ from the LASSO model using a theta power band provides the best performance prediction at 74.6% with contributions from 48 EEG channels. Thus, we conclude that, for the addition task, we use alpha brain power from 51 EEG channels, and for the typing task, we use theta brain power from 48 EEG channels as features for performance predictions.

Furthermore, based on the LASSO models obtained for the two office-work tasks we statistically determined the reliability of performance prediction induced by two indoor temperatures with alpha power (i.e., addition task) and theta power (i.e., typing task). In other words, if the residual errors between observed and predicted performance follow a random normal distribution, this indicates that the LASSO model has considered all features in linear regression analyses towards predicting performance. To do so, we use the *t*-test on the error values from both exposures for each task. The null hypothesis for the t-test being performance errors induced by the two room temperatures are the same. *p*-values of 0.7756 and 0.5605 were obtained for the addition and the typing task. At a significance level α = 0.1, the tests failed to reject the null hypothesis of equal performance error means, implying that changes in performance are sufficiently explained by the features selected by the LASSO technique.

#### 3.3.3. Prediction of Performance

Finally, we investigated the power of using brain power bands as regressors for predicting performance and compared them to other physiological signals, that is, skin temperature and heart rate. For physiological signals, polynomial models of model orders 1–9, were fitted by using the same data as used for LASSO. Table 6 shows the mean squared errors (MSE) of all biomarkers presented in this paper. It is not surprising to find that the LASSO predictors using PSDs obtained much smaller MSEs than those from skin temperature and heart rate (even with a higher order polynomial model). Taken together, these results confirm that neurophysiological signals recorded using EEG are better predictors of human performance induced by different indoor room temperatures. 

#### 3.3.4. Brain Activity Pattern

**Figure 3:**
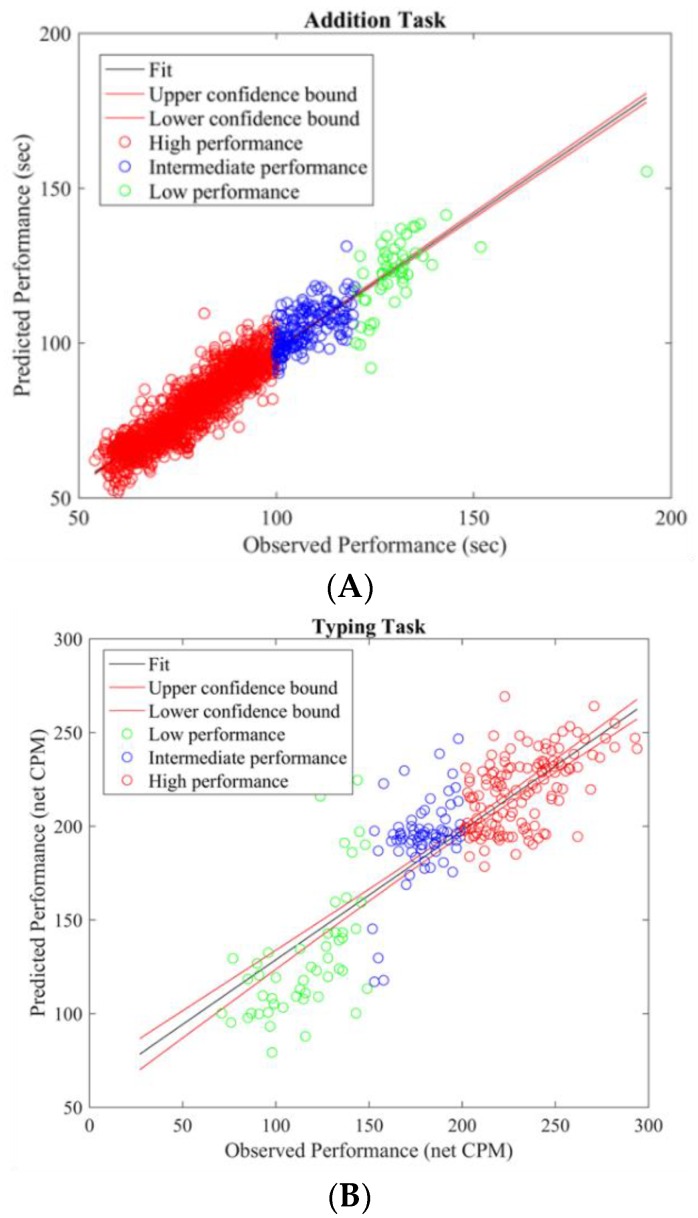
(**A**) Addition task—scatter plot of observed versus predicted performance (seconds) by using LASSO linear regression model with alpha power as a single regressor; (**B**) Typing task—scatter plot of observed versus predicted performance (net CPM) by using LASSO linear regression model with theta power as a single regressor.

**Figure 4:**
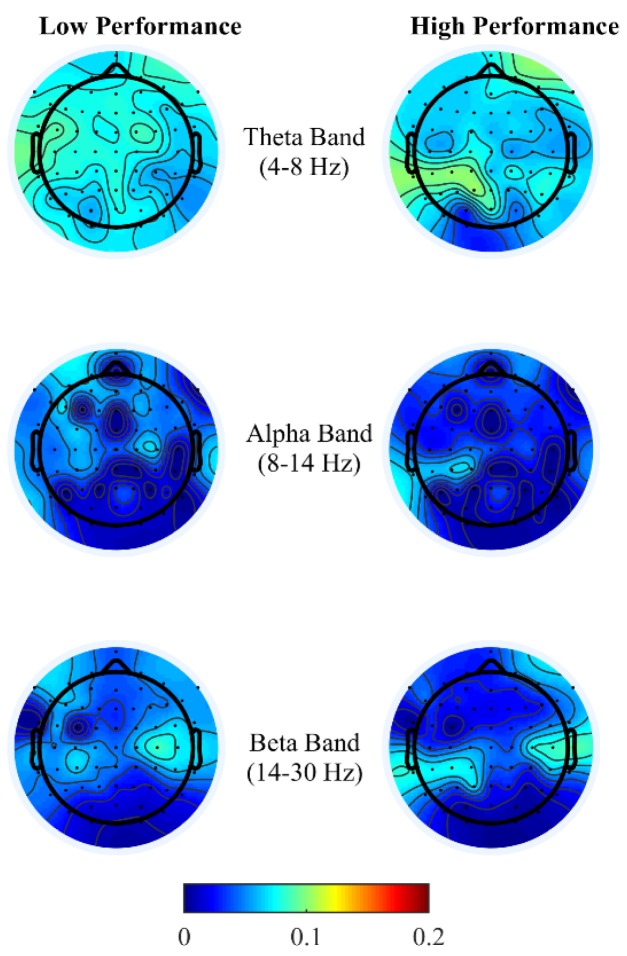
Scalp activities across EEG channels with non-zero LASSO coefficients from brain spectral power for low- and high-addition task performance.

**Figure 5:**
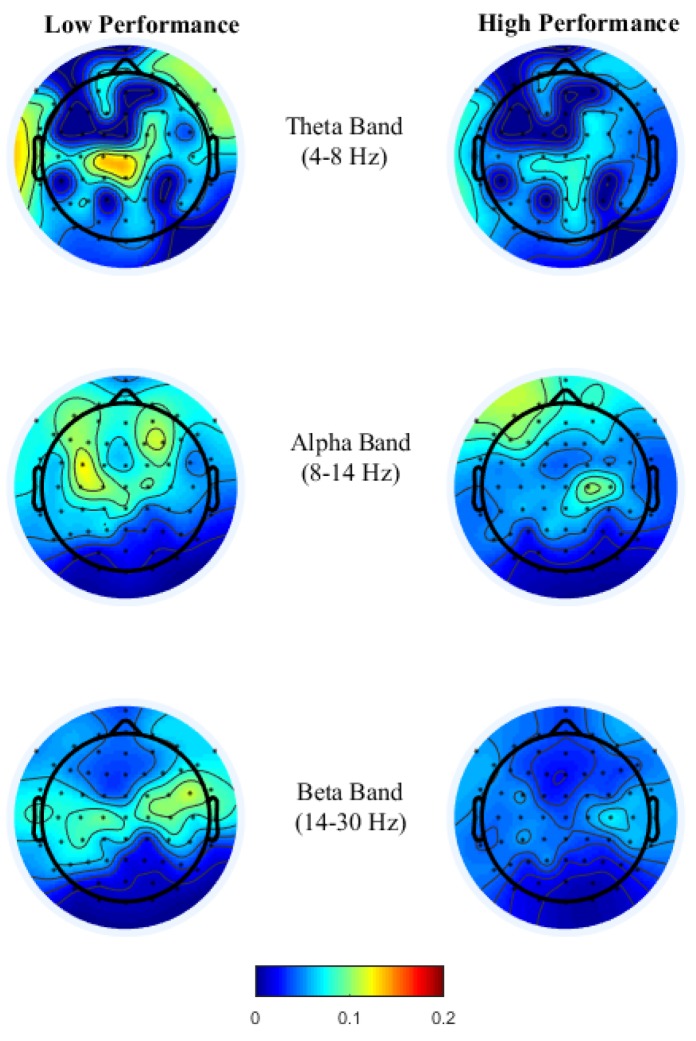
Scalp activities across EEG channels from non-zero LASSO coefficients from brain spectral power for low- and high-typing task performance.

To gain insight into the mental functions that are induced by varying thermal conditions during these office-work tasks, we investigate the differences in brain activity patterns arising from change in performance from all power bands. To do so we categorized participants’ performance (i.e., API and net CPM) into two groups, low- and high-performance by defining cutoff values based on the scatter plots obtained between observed and predicted performance using the LASSO model as seen in Figure 3A,B. For the addition task’s performance cutoff values, samples less than 100 s were labeled as high performance and values greater than 120 s were labeled as low performance. Likewise, for the typing task, samples less than 100 net CPM were labeled as low performance and those greater than 150 CPM were labelled as high performance. Based on these cutoff values, we plot the average brain activities on the scalp projected from EEG channel locations with non-zero coefficients obtained from the LASSO model from each brain power band, shown in Figure 4 and Figure 5. 

Brain activity patterns from both office-work tasks over low and high performance may be interpreted as the average spatial temporal brain power density patterns over a time window corresponding to the performance index of the specific task, that is, in the addition task, over the time taken to answer 20 questions correctly and for the typing task, net characters typed per minute. For the addition task, see Figure 4, congruent with the findings from References [16,17,18,19,20,21] we observe high localized theta power over the right prefrontal and left parietal cortex during high performance than compared to low performance. This is indicative of the frontal-parietal network being associated with working memory and executive functions of arithmetic problem-solving. High theta activity at the right frontal cortex reflects arithmetic fact retrieval, while differential brain temporal dynamics across the frontal-parietal cortex reflect higher cognitive demands for multistep procedural strategies. Since frontal theta activity is common in retrieving basic arithmetic facts, LASSO was unable to pick this feature as most discriminant. Also, bilateral beta activity, particularly during high performance, is reported to be associated with the conceptual processing of numbers, that is, the identification of operands in the addition task [43,44]. Although in this study design we are unable to conclusively compare arithmetic problems between retrieval or multistep procedures based on the subjective difficulty levels, we can infer that discomfort due to thermal conditions created demands of higher cognitive load to either maintain the current performance level or to achieve high performance. Overall, in agreement with the findings from Reference [16], desynchronization of alpha and beta power is lower at the frontal and parietal region, with right frontal theta enhancement reflecting distinct cognitive functions in multicomponent problem solving.

High performance in the typing task requires both motor control with sustained attention, a similar psychological requirement to brain oscillations in sports activities. As theorized earlier in the typing task, see Figure 5, theta power in the fronto-midline is found to be the most discriminant feature for performance prediction wherein high theta is observed during low performance at the frontal-midline and the second most discriminant feature is frontal alpha event-related desynchronization (ERD) during high performance. Studies by References [27,28,29] have associated high frontal theta and high parietal alpha power differentiating skilled sports players to novices, reflecting developed task solving strategies, focused attention and an economic parietal sensory information processing. The results found in this paper for the typing task observed an opposite trend in the theta band at different performance levels, as the typing task involved reinforcement learning where subjects were required to rectify typing errors in order to proceed, forcing subjects to refocus and retype. Thus, enhanced frontal-midline theta power during low performance in our data possibly reflects error feedback information processing and subsequently increasing response time to retype correctly reflected as high beta power at the somatosensory cortex during low performance. Alpha activity amplitudes have been shown to be inversely related to the amount of neuronal population activated during cognitive-motor tasks. Studies by References [45,46,47,48] have related alpha and beta ERD for skilled performers to be associated with fine cognitive-motor performance. This is consistent with our findings, where during high performance alpha and beta ERD were observed over premotor and sensorimotor areas reflecting confidence in typing correctly, which requires precise planning and regulation of bilateral finger movements.

Overall, brain activity patterns presented in Figure 4 and Figure 5 show that during high performance there is lower activity than during low performance, which is almost in line with the “neural efficiency” hypothesis. These brain activity patterns enable the creation of a unique EEG profile for varying degrees of performance level, which are task-dependent. In this study, we use EEG sensor space features to predict performance, which are limited by volume conductance, while it is possible that source space estimates could provide better predictions. Additionally, it is possible that functional connectivity estimates at source level could provide higher prediction ability as reported in Reference [49]. However, these are the current two limitations of our study as we analyze data in EEG sensor space only and treat changes in brain states as a continuous task rather than ‘event related.’ The main motivation to conduct analyses in this fashion is to use this analysis technique in real-time brain computer interfaces in a realistic office-work setting to predict environmental conditions based on performance predictions from EEG power spectral densities.

## 4. Conclusions

To the best of our knowledge, we are the first to present preliminary results of using EEG power spectral densities to predict performance changes due to change in indoor-room temperature. Our analysis statistically validates that office-work performance is impacted by varying indoor temperature. We present a comprehensive regression analysis for predicting performance in two different office-work tasks using features reported by past studies and neurophysiological EEG signals. We found that EEG brain band power is the best predictor of performance, which was enhanced using the LASSO regression technique. This method found alpha brain power to be the best feature corresponding to the right frontal and left parietal cortex for the arithmetic problem-solving task (*R*^2^ = 88.6%) and theta brain power as the best feature corresponding to the fronto-middle cortex for the typing task (*R*^2^ =74.6%). Lastly, the robustness of using EEG power spectral densities as features was reported by mean-square errors. With LASSO, we were able to achieve performance prediction abilities five times greater than using a single linear regression model and 17 times higher prediction ability than compared to using thermal survey votes, skin temperature and heart-rate. While the results of this study are promising, there are a few limitations. Currently, we are unable to confirm the behavioral trend, that is, whether there is an increase or decrease in performance under different indoor temperatures due to insufficient population size, which is why we report prediction strength using linear regression analysis and were still able to achieve promising results. Also, it is possible that upon collection of data from more subjects, the relationship between EEG power spectral densities and office-work performance under different thermal conditions may not be linear, thereby a non-linear regression technique, or other machine learning techniques may be needed for classifications. In the future, this research needs to focus on more comprehensive investigations of performance under longer exposures and using varying workloads and further methodological studies are needed to investigate prediction models to classify cross-task performance. Ultimately, this domain of research aims to provide motivation for future research to help achieve optimal productivity from office-workers by providing feedback regarding their environmental conditions. 

## Figures and Tables

**Table 1:** Kolmogorov-Smirnov (KS) test results on addition task performance under two indoor temperatures. Columns 2 & 3 show the average task performance with standard deviation in parenthesis. Column 4 shows the *p*-values from the statistical test.

Subject	22.2 °C (72 F)	30 °C (86 F)	KS-Test (*p*-Value)
S1	86.9 (±8.5)	101.9 (±18.8)	7.2741 × 10^−15^
S2	75.9 (±5.5)	70.1 (±8.6)	3.8359 × 10^−15^
S3	85.9 (±13.3)	87.0 (±7.5)	0.0051
S4	69.5 (±7.8)	64.5 (±5.3)	3.7328 × 10^−11^
S5	99.7 (±9.9)	90.0 (±6.5)	1.2082 × 10^−18^
S6	73.5 (±8.5)	78.6 (±7.9)	6.4751 × 10^−11^
S7	90.1 (±11.9)	93.5 (±15.3)	0.0021

**Table 2:** KS test results on typing task performance under two indoor temperatures. Columns 2 & 3 show the average performance with standard deviation in parenthesis. Column 4 shows the *p*-values from the statistical test.

Subject	22.2 °C (72 F)	30 °C (86 F)	KS-Test (*p*-Value)
S1	185.25 (±27.3)	207.5 (±25.9)	0.0186
S2	121.5 (±18.8)	122.17 (±34.1)	0.1687
S3	240.6 (±22.8)	226.7 (±30.8)	0.0875
S4	199.7 (±26.2)	214.5 (±16.2)	0.0076
S5	104.5 (±27.9)	120.5 (±19.3)	0.0420
S6	178.3 (±37.3)	191.3 (±26.9)	0.1687
S7	228.0 (±28.1)	244.9 (±37.3)	0.0420

**Table 3:** Correlation *R*^2^ between simulated office-work performance and different physiological predictors.

*R* ^2^	Thermal Sensation	Thermal Comfort	Skin Temperature	Heart Rate
Addition Task	0.00369	0.018	0.0127	0.0089
Typing Task	0.0714	0.104	0.0201	0.052

**Table 4:** Correlation coefficients (*ρ*) between brain band pairs corresponding to the EEG electrodes with highest *R*^2^ single regressor linear models.

Single Regressors	Correlation Coefficient (*ρ*)	Addition Task	Typing Task
Theta Band	Theta–Alpha	0.6978	0.4380
	Theta–Beta	0.6663	0.5549
Alpha Band	Alpha–Theta	0.6788	0.3862
	Alpha–Beta	0.6303	0.6130
Beta Band	Beta–Theta	0.7048	0.3065
	Beta–Alpha	0.6303	0.5176

**Table 5:** *R*^2^ obtained between observed and estimated performance for office tasks using LASSO regression and the number of non-zero coefficients in the fitted LASSO model.

*R* ^2^	Theta Band (4–8 Hz)	Alpha Band (8–14 Hz)	Beta Band (14–30 Hz)	Combined Bands
**Addition Task**	0.681	0.886	0.67	0.834
(# non-zero coefficients)	(#64)	(#51)	(#62)	(#174)
*p*-values on fitting	0	0	0	0
**Typing Task**	0.746	0.712	0.696	0.645
(# non-zero coefficients)	(#48)	(#38)	(#43)	(#45)
*p*-values on fitting	3.5292 × 10^−91^	7.2004 × 10^−86^	1.054 × 10^−84^	1.6241 × 10^−25^

**Table 6:** MSE obtained from LASSO model using brain PSDs & from polynomial curve fitting models using physiological signals.

Brain Band	Mean Square Errors	
	Addition Tasks	Typing Tasks
Theta (4–8 Hz)	79.97	600.42
Alpha (8–14 Hz)	27.55	682.48
Beta (14–30 Hz)	79.15	717.30
Combined Bands	40.15	1127.30
Skin temperature	2612.5 (6th order)	37002 (5th order)
Heart Rate	284.5460 (7th order)	33361 (4sh order)
